# Management of Infected Tissues Around Dental Implants: A Short Narrative Review

**DOI:** 10.1590/0103-6440202406160

**Published:** 2024-10-25

**Authors:** Hamdan Alghamdi, Minas Leventis, Tatiana Deliberador

**Affiliations:** 1 Department of Periodontics and Community Dentistry, College of Dentistry, King Saud University, Riyadh, Saudi Arabia. H.A; 2 Department of Oral and Maxillofacial Surgery, Dental School, National and Kapodistrian University of Athens, Greece; 3 Latin American Institute of Dental Research and Education - ILAPEO, Curitiba/PR, Brazil

**Keywords:** peri-implantitis, dental implants, biofilm, antibacterial, surface engineering

## Abstract

Dental implants have become the most effective treatment option for replacing missing teeth, worldwide. The popularity and demand for dental implants are continually increasing. Nevertheless, its complications are undeniable. Peri-implant diseases, including peri-implant mucositis and peri-implantitis, are a multifaceted clinical condition. Therefore, it is in the best interest to optimize the management of peri-implantitis, and there are still numerous methods to treat and manage infections in the vicinity of dental implants. The main goal of peri-implantitis treatment is to arrest disease progression, eliminate infection, and reconstruct damaged tissues around the implant. The clinical evidence on treating peri-implantitis that is available in PubMed was reviewed. Additionally, we presented the most comprehensive management strategies. As a result, numerous clinical trials recommended mechanical debridement and local administration of antimicrobial agents as well as topical oxygen therapy to mitigate bacterial biofilm and manage infection. The regenerative (bone grafting) approach for the treatment of peri-implantitis is another effective method. Finally, implant surface engineering can address high antibacterial efficacy and site-specific biofilm reduction.

## Resumo

Os implantes dentários tornaram-se a opção de tratamento mais eficaz para a substituição de dentes perdidos em todo o mundo. A popularidade e a demanda por implantes dentários estão aumentando continuamente. No entanto, suas complicações são inegáveis. As doenças peri-implantares, incluindo mucosite peri-implantar e peri-implantite, são condições clínicas multifacetadas. Portanto, é de grande interesse otimizar o manejo da peri-implantite. Ainda existem vários métodos para tratar e gerenciar infecções nas proximidades dos implantes dentários. O principal objetivo do tratamento da peri-implantite é interromper a progressão da doença, eliminar a infecção e reconstruir os tecidos danificados ao redor do implante. Foi revisada a evidência clínica disponível no PubMed sobre o tratamento da peri-implantite. Além disso, apresentamos as estratégias de manejo mais abrangentes. Como resultados, diversos ensaios clínicos recomendaram o debridamento mecânico e a administração local de agentes antimicrobianos, bem como a terapia tópica com oxigênio para mitigar o biofilme bacteriano e controlar a infecção. A abordagem regenerativa (enxerto ósseo) para o tratamento da peri-implantite é outro método eficaz. Finalmente, a engenharia de superfícies de implantes pode abordar a alta eficácia antibacteriana e a redução do biofilme de forma específica no local.

**Figure:**
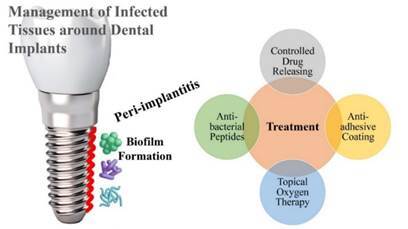

## Introduction

Globally, approximately 5 million dental implants are placed annually ^1^. This number is expected to increase as more individuals require oral care treatment. In reality, dental implant therapy has emerged as the most effective intervention for replacing missing teeth and esthetics ^2^. However, the placement of dental implants is not without the potential of complications. For instance, the most prevalent risk of infection is generally associated with peri-implant tissues ^2^. Unfortunately, dentists and patients are confronted with many clinical and financial challenges as a result of peri-implant diseases. 

**Figure 1:**
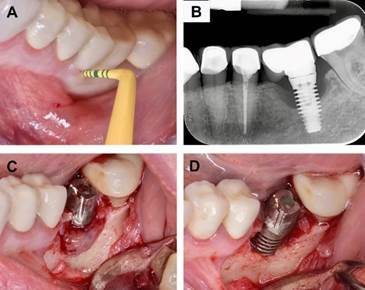
A, patient was admitted to the clinic with peri-implantitis in the #36 region. Inflammation and profound peri-implant probing depths were observed at the site. B, the mesial and distal aspects of the implant exhibited peri-implant bone loss in a periapical radiograph. C, the prosthetic crown was removed and a surgical incision was opened to provide complete access to the peri-implant defect. The site was subsequently treated using the following protocol, D: the removal of granulation tissue, the instrumentation of the implant surface with titanium curettes, and the application of blue-m oral gel (BlueM Europe, Zwolle, The Netherlands) twice for a duration of 5 minutes. Normal saline was then used to rinse the implant surface after each application. Ultimately, resective surgery was implemented, including osteoplasty and ostectomy.

In general, there are two primary categories of peri-implant diseases ^3^ ^,^ ^4^. Initially, peri-implant mucositis is a condition that affects the mucosa surrounding dental implants, similar to gingivitis. Peri-implant mucositis is a reversible inflammation with clinical signs of redness, swelling, and bleeding upon probing ^5^. Secondly, peri-implantitis is comparable to periodontitis. It is characterized by loss of bone, and infection in the vicinity of the implant ^6^. Figure 1 illustrates peri-implantitis as a serious condition that is marked by pocket formation, destruction of supporting bone, formation of granulation tissues, suppuration (pus formation). Additionally, it has the potential to result in implant failure.

Both peri-implant diseases are attributed to poor oral hygiene, smoking, medical conditions, and inadequate implant maintenance ^4^. Consequently, it is crucial that the diagnosis and prompt treatment of peri-implant diseases are advantageous to both patients and dental clinics. The objective of this short narrative review is to integrate the evolving concepts in the treatment of peri-implant diseases. In addition, the ultimate goal of our concise review is to concentrate on the clinical recommendations that are supported by evidence, as well as to identify voids in knowledge and direct future research on this subject.

### Pathophysiology of peri-implant diseases

Infected tissues around dental implants are primarily associated with the invasion of many microorganisms ^7^. Mainly, the pathogenic bacteria form biofilms on the implant surface and surrounding tissues often contain a mix of gram-negative anaerobic bacteria, similar to those found in periodontal disease ^8^ ^,^ ^9^. Recent researchers have identified common pathogens include *Porphyromonas gingivalis*, *Treponema denticola*, *Tannerella forsythia*, and *Fusobacterium nucleatum* ^10^.

Inside the biofilm, pathogenic bacteria are growing fast and become more resistant to the host's immune response ^11^. In addition, the bacteria biofilms can penetrate into the surrounding soft tissue and bone, leading to inflammation, infection, and tissue destruction ^10^ ^,^ ^11^. Other microorganisms, such as fungi and viruses, can also potentially contribute to peri-implant infections, although bacterial infections are the most commonly studied ^11^.

Peri-implant biofilms ensure bacterial cells a long-term survival by several mechanisms ^12^. First, biofilms are providing a physical barrier that limits immune cell penetration, thereby preventing phagocytosis killing ^13^. Additionally, the bacterial biofilms can exhibit varying growth rates, altered oxygen and nutrition requirements, and acquired virulence mechanisms ^13^. Because of these factors, bacteria found in biofilms develop a strong resistance to antimicrobial therapy up to 1000 times higher than free-swimming bacteria ^14^. 

The bacterial biofilm infections have been shown to elicit bone resorption and cause immense damage to the surrounding host tissue ^13^. Due to the infection of bone around the dental implant, PAMPs interact with innate toll-like receptors (TLRs) and then stimulate the release of inflammatory cytokines such as TNF, IL-1, and IL-6 ^12^ ^,^ ^13^ ^,^ ^15^. As a result, osteoclasts get activated, and osteoblasts produce more RANKL, which causes the RANKL/OPG ratio to change in favor of bone resorption ^15^. Furthermore, microbiological studies have shown that some bacterial species are capable of damaging bone tissue, independently.

Basically, the first step in the creation of biofilms is the microorganism's adsorption occurs at the acquired pellicle on the implant surface ^9^ ^,^ ^16^. This process is facilitated by hydrophobic, electrostatic, and van der Waals interactions, which permit unspecific attachment. Following the initial colonization, bacteria start to create a layer of extracellular polymeric substance (EPS), called biofilm, and made up of proteins, lipids, exopolysaccharides, and nucleic acids ^16^. Biofilm maturation involves a high growth of bacterial density and production of virulence factors, which is associated with chronic infections. Inside a mature biofilm, the quorum sensing (QS) signaling system mediates all communication between bacterial cells ^16^. By QS signaling, the pathogenic bacteria can survive in the hostile environment using different adaptation mechanisms ^16^.

In summary, bacterial biofilms pose a serious threat to peri-implant tissues. A comprehensive strategy that incorporates preventive measures and cutting-edge treatment approaches to effectively tackle biofilm-associated infections is needed.

### Evolving concepts in treatment of peri-implant diseases

The main goal of peri-implantitis treatment is to arrest disease progression, eliminate infection, and reconstruct damaged tissues around the implant ^17^. The regeneration of the peri-implant hard tissues depends on several factors. A tentative regenerative procedure includes the application of various grafting materials and/or resorbable membranes ^17^.

Based on PubMed search using “Peri-implantitis” keyword, the results were more than 4000 Title/Abstract. By limiting the results of the search to Clinical Trials, we were able to include 221 articles for review and assessment (unpublished data). Inclusion criteria: no restrictions were applied on study design included in each clinical trial; the characteristics of patients, the number of dental implants and type of prosthetic restorations, and type of peri-implantitis treatment (nonsurgical or surgical). Exclusion criteria: non-English language studies and self-reported peri-implant status were excluded.

The previous clinical data recommended a variety of treatment options for peri-implantitis, which may differ based on the severity of the condition. In general, certain authors suggested that non-surgical therapy should involve mechanical debridement of the infected tissues and implant surface to eliminate biofilm and calculus. Subsequently, numerous clinical trials recommended the local administration of antimicrobial agents (e.g., chlorhexidine, antibiotics) to mitigate bacterial load and manage infection ^17^ ^,^ ^18^.

### Non-surgical therapy (mechanical debridement)

In fact, non-surgical debridement is frequently the initial method employed to address infected tissues in the vicinity of dental implants ^18^. This procedure typically entails the comprehensive removal of debris, plaque, and calculus from the implant surface and the adjacent tissues. Its objective is to reduce inflammation and bacterial infection without necessitating invasive procedures. Nevertheless, in certain instances of advanced or persistent infection, surgical intervention may be required to completely access and clean the area surrounding the implant ^17^. Manual instruments and ultrasonic devices are the primary tools and devices used in the clinical setting for the non-surgical mechanical debridement ^18^. Air particle abrasive systems (e.g., air-polishing) can be employed in conjunction with instrument cleaning to eliminate biofilm and calculus from difficult-to-reach regions surrounding the implant. The efficacy of these instruments and devices in managing peri-implant diseases has been the subject of numerous RCTs. These mechanical debridement methods have been demonstrated to decrease peri-implant inflammation, as evidenced by the plaque and bleeding-on-probing scores. Nevertheless, there was no discernible effect on the probing depth (PD) ^18^.

### Antibiotics

In cases where the infection is severe or has spread beyond peri-implant tissues, the administration of local antibiotics can be a critical component of the treatment strategy for peri-implantitis ^19^. 

Given that the biofilm of peri-implantitis contains a broader spectrum of pathogenic microorganisms, it may be even more crucial to reduce the number of pathogens and alter the bacterial biofilm composition in the management of peri-implantitis. From this perspective, the administration of antibiotics could be advantageous for the management of biofilm dysbiosis. In 1992, Mombelli and Lang were the first to recommend the use of antibiotics as adjuncts to peri-implantitis treatment ^20^.

Several studies have been conducted since that time to assess the beneficial effects of systemically and locally delivered antibiotics in conjunction with other interventions. Nevertheless, the efficacy of concurrent systemic or local antibiotic administration in the management of peri-implantitis remains a matter of debate. Furthermore, the use of antibiotics should be appropriately assessed, as they are frequently prescribed by dentists without specific indications, thereby violating antibiotic stewardship.

Feres et al. ^21^ recently endeavored to critically evaluate the literature on the use of antibiotics to treat peri-implantitis in order to unravel the effectiveness of antibiotics for peri-implantitis treatment. The ultimate objective was to support evidence-based clinical recommendations, identify gaps in knowledge, and guide future studies on this topic. There is insufficient data to substantiate a specific evidence-based antibiotic protocol for the treatment of peri-implantitis through surgical or nonsurgical therapy; however, certain conclusions may be derived. An effective protocol for enhancing the results of nonsurgical treatment is the use of systemic metronidazole (MTZ) as an adjunct to ultrasonic debridement. The clinical and microbiological effects of amoxicillin (AMX) as adjuncts to optimal nonsurgical implant decontamination protocols or open-flap debridement should be evaluated in potential future studies. Furthermore, RCTs should evaluate antibiotic-loaded surfaces and new locally delivered medicines.

Nevertheless, antibiotic resistance can be exacerbated by the inappropriate use of antibiotics, which could potentially impact the long-term outcomes of peri-implant treatment ^19^. Consequently, antibiotics should be employed with caution and as part of a comprehensive treatment regimen. 

### Chlorhexidine

Chlorhexidine is frequently employed in periodontology due to its broad-spectrum antimicrobial properties ^22^. For dental implants, many clinicians used to prescribe antiseptic mouthwash to patients as part of their daily oral hygiene regimen. This could potentially mitigate the risk of peri-implant diseases by preventing the accumulation of bacterial plaque on implant surfaces and adjacent gingival tissues ^23^. Nevertheless, chlorhexidine primarily acts on the surface of tissues and is unable to efficiently penetrate deep into the bacterial biofilms.

In cases of peri-implantitis, the clinical benefits of chlorhexidine are questionable ^24^. This antiseptic compound, like antibiotics, could result in an increase in resistant bacteria and a reduction in the therapeutic efficacy ^24^. In addition, the bacterial biofilm is encased in a protective exopolysaccharide material, which prevents antiseptic molecules from accessing biofilm and then enhances bacterial tolerance ^24^. Consequently, it is necessary to target both the biofilm matrix and the specific microbial cells within the pathogenic biofilm. Further, research has demonstrated that chlorhexidine solutions have a cytotoxic effect on peri-implant tissue cells (e.g., fibroblasts, osteoblasts, and lymphocytes), which has the potential to impede the wound healing process ^25^.

### Topical oxygen therapy (hydro-carbon-oxo-borate complex - HCOBc)

In recent years, many research institutes have developed alternative therapies to chlorhexidine ^26^. For instance, topical oxygen therapy, using active oxygen complexes, is a novel treatment approach to promote healing and combat infections ^26^. Although research on the efficacy of topical oxygen therapy in the context of peri-implantitis is still in the early stages, it has been investigated as a potential adjunctive therapy ^27^. The objective of oxygen therapy is to establish an oxygen-rich environment that encompasses the peri-implant tissues. This has the potential to promote tissue repair, enhance tissue oxygenation, and inhibit the proliferation of anaerobic bacteria ^27^.

**Figure 2:**
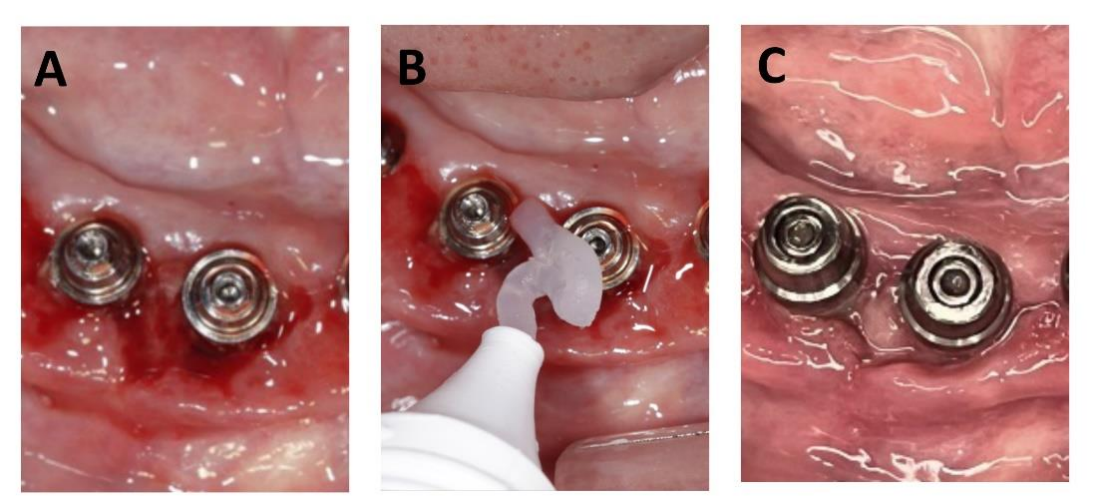
A**,** an inflamed peri-implant tissue was detected after removal of prosthesis. Bleeding on probing was scored high. Probing depth: 2 to 4 mm. Diagnosis of peri-implant mucositis. **B,** non-surgical mechanical debridement was done with application of oxygen blue®m gel. **C**, one-week follow-up showed healthy appearance of the peri-implant tissues.

Recently, a Dutch research group has developed a chemical complexation of hydro-carbon-oxo-borate, called active-oxygen complex (BlueM Europe, Zwolle, The Netherlands) ^26^. Interestingly, this formula provides controlled release of active oxygen without generating hydroxyl radicals ^26^. Furthermore, the active oxygen formula has the potential to employ antimicrobial mechanisms that are distinct from those of other conventional antiseptic agents. It has the potential to selectively inhibit microbial biofilm composition and maturation toward a pathogenic status ^27^ ^,^ ^28^. In a recent study conducted by Shibli et al. ^28^, the active oxygen formula exhibited an extraordinary selective effect on the most periodontal pathogenic bacteria (known as the red complex). Clinically, the active oxygen formula is highly effective in inhibiting the colonization and proliferation of pathogenic biofilms that are linked to the majority of oral infections, including periodontitis, and peri-implant diseases (Figure 2). Additionally, Ntrouka et al. ^29^ verified that a product containing an active-oxygen complex was capable of eliminating substantially more *S. mutans* that were present as polymicrobial biofilms on the titanium surface in vitro. An in vivo evaluation of mature oral biofilm was conducted by Mostajo et al. ^30^ after a seven-day cleansing treatment with mouthwash that contained an active oxygen formula. The biofilm was collected from healthy young adults. The results of this study indicate that this product has the potential to selectively inhibit oral bacteria, albeit in a clinically significant manner. Following a seven-day rinsing treatment period, changes in oral microbiome populations were observed.

Although there is some evidence to support the use of oxygen therapy in the treatment of other types of infections and lesions, clinical studies that specifically evaluate topical oxygen therapy for peri-implantitis are limited and frequently conducted on a small scale ^26^. In order to determine its efficacy, optimal protocols, and long-term advantages in the management of peri-implantitis, additional research is required ^27^.

### Surgical approaches

The treatment of peri-implantitis requires surgical intervention to eliminate the pocket, clean the infected defect, and decontaminate the implant surface ^17^. The progression of peri-implantitis can be effectively treated by resective surgery (i.e., ostectomy and osteoplasty) in combination with the smoothing and polishing of the dental implant surface ^31^. However, the resective approach is associated with increased postoperative recessions, which is not suitable for esthetic-sensitive areas. Consequently, the regenerative approach for the treatment of peri-implantitis is another effective surgical technique, as recommended by numerous recent clinical studies ^32^. Mainly, the application of guided bone regeneration (GBR) with various grafting materials and/or resorbable membranes resulted in favorable therapeutic outcomes ^32^. GBR procedures appear to be a predictable treatment for peri-implant defects ^17^. 

Nevertheless, researchers and clinicians have different opinions on using surgical intervention, and the best possible approach is not very clearly described. According to a recent systematic review ^17^, clinical parameters such as BOP and PPD, demonstrated a superior improvement in the surgical treatment using bone graft materials. However, the optimal surgical treatment procedure remains contingent upon the clinical presentation and the severity of peri-implantitis defects.

### Surface engineering to prevent peri-implant diseases

**Figure 3:**
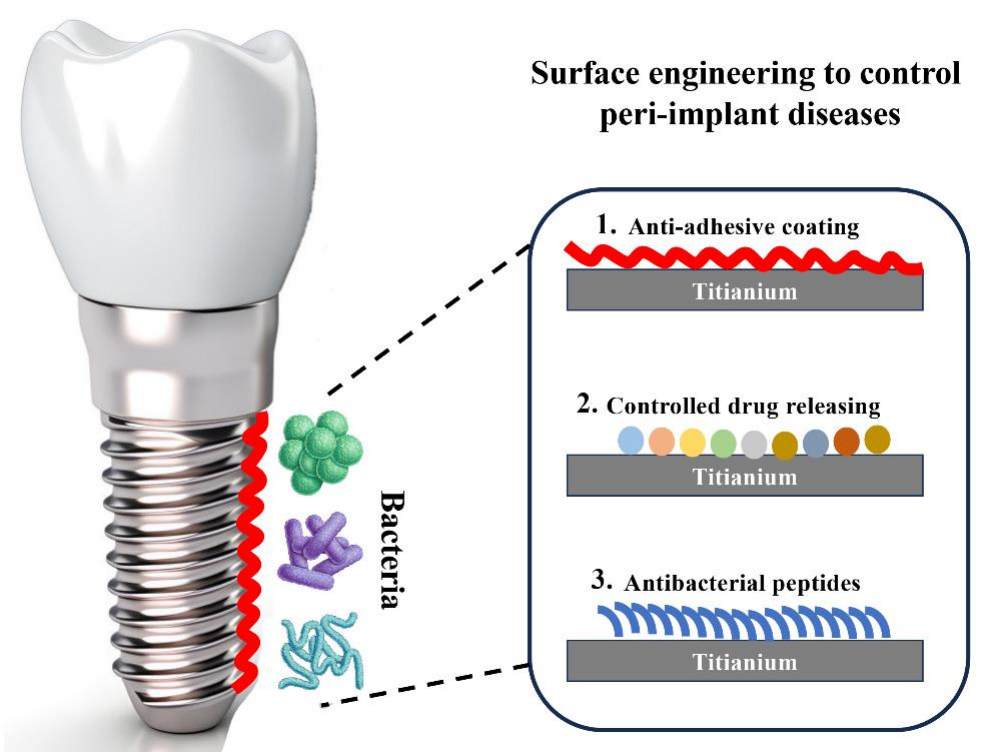
An illustration shows advanced surface modifications and coatings that can be utilized with several methods to prevent bacterial adhesion and biofilm formation on implant surfaces. Second, surface coating with controlled drug release to kill the bacteria without being cytotoxic to human cells. Third, implant surfaces can be functionalized with antibacterial peptide antibiotics to prevent/reduce peri-implant diseases.

To maintain a healthy peri-implant tissue, surface engineering with antimicrobial properties has been proposed to prevent bacterial adhesion and biofilm formation ^33^. A common approach is the surface coatings with antimicrobial agents to kill the bacteria without being cytotoxic to human cells ^33^. As in Figure 3, advanced surface modifications and coatings can be utilized with several methods including plasma spraying, electrospinning, sol-gel, hydrothermal method, and covalent immobilization (34). 

Some researchers have suggested different nanoparticles and ions (e.g., Cu, Ag, Au, and Zn) as examples of antibacterial agents (34). Surface-loaded with antibiotics showed also high efficacy against peri-implant pathogens, *in vitro* (34). Recently, scientists have engineered surfaces with infection-targeted antimicrobial ability (34). These types of surfaces can address high antibacterial efficacy and site-specific biofilm reduction. Another laboratory study to control peri-implant infection is by modifying surfaces with an anti-adhesive topography (34). Such texture inhibits bacterial adhesion and growth onto the implant surface (34). Furthermore, implant surfaces can be bactericidal by repelling the bacteria due to hydrophobic properties. However, the *in vitro* research on antibacterial surface engineering is still not ready for clinical translation due to technology complexity ^33^ ^,^34). 

## Conclusions

For both patients and clinicians, peri-implantitis is an unfortunate complication. We have provided a comprehensive and current analysis of the clinical evidence concerning the management of peri-implantitis in this review. It is certain that the management of peri-implantitis will be subjected to the development of biomaterials and clinical modalities. The selection of treatment methods is based on the clinical presentation and the severity of peri-implantitis defects. Nevertheless, the utilization of a combination therapy that incorporates both surgical and non-surgical methods appears to be more suitable for the treatment of peri-implant diseases. Additionally, there is potential for the development of more robust experiments through research on surface engineering with antibacterial properties. Additionally, research on the prevention of peri-implant infection will be instrumental in alleviating the overall burden of peri-implantitis.
